# Friend or Foe? Essential Roles of Osteoclast in Maintaining Skeletal Health

**DOI:** 10.1155/2020/4791786

**Published:** 2020-03-03

**Authors:** Haixing Wang, Guangpu Yang, Yinbo Xiao, Guotian Luo, Gang Li, Ziqing Li

**Affiliations:** ^1^Department of Orthopaedics & Traumatology, Faculty of Medicine, The Chinese University of Hong Kong, Prince of Wales Hospital, Hong Kong, China; ^2^Stem Cells and Regenerative Medicine Laboratory, Li Ka Shing Institute of Health Sciences, The Chinese University of Hong Kong, Prince of Wales Hospital, Hong Kong, China; ^3^Centre for the Cellular Microenvironment, Institute of Molecular, Cell & Systems Biology, College of Medical, Veterinary and Life Sciences, University of Glasgow, Glasgow, UK; ^4^Université de Paris, CNRS, INSERM, B3OA, Paris, France; ^5^Ecole Nationale Vétérinaire d'Alfort, B3OA, Maisons-Alfort, France; ^6^Department of Anatomy and Cell Biology, School of Dental Medicine, University of Pennsylvania, Philadelphia, PA, USA; ^7^Department of Basic and Translational Sciences, School of Dental Medicine, University of Pennsylvania, Philadelphia, PA, USA

## Abstract

Heightened activity of osteoclast is considered to be the culprit in breaking the balance during bone remodeling in pathological conditions, such as osteoporosis. As a “foe” of skeletal health, many antiosteoporosis therapies aim to inhibit osteoclastogenesis. However, bone remodeling is a dynamic process that requires the subtle coordination of osteoclasts and osteoblasts. Severe suppression of osteoclast differentiation will impair bone formation because of the coupling effect. Thus, understanding the complex roles of osteoclast in maintaining proper bone remodeling is highly warranted to develop better management of osteoporosis. This review aimed to determine the varied roles of osteoclasts in maintaining skeletal health and to highlight the positive roles of osteoclasts in maintaining normal bone remodeling. Generally, osteoclasts interact with osteocytes to initiate targeted bone remodeling and have crosstalk with mesenchymal stem cells and osteoblasts via secreted factors or cell-cell contact to promote bone formation. We believe that a better outcome of bone remodeling disorders will be achieved when proper strategies are made to coordinate osteoclasts and osteoblasts in managing such disorders.

## 1. Introduction

Bone is a dynamic organ that continuously remodels in a well-orchestrated manner to support body-required mechanical characteristics and maintain calcium homeostasis throughout one's lifetime [1, 2]. This constant remodeling process requires delicate coordination from multiple cell types, in which hematopoietic stem cell- (HSC-) derived osteoclast (OC) lineage and bone marrow mesenchymal stem cell- (BMSC-) derived osteoblast (OB) lineage receive the most attention [3–5]. Balance between bone resorption by OCs and bone formation by OBs is usually maintained during the physiological process but dies away under pathological conditions, such as inflammation, diabetes, aging, and cancer, resulting in bone remodeling-related disorders and diseases, such as osteoporosis, periodontitis, inflammatory arthritis, Paget's disease, or tumor-induced osteolytic bone metastasis [6–10]. OCs, the giant cells that are responsible for bone removal in the skeletal family, have always been considered to be the main culprit in these disorders and diseases because of its overactive functionalities under pathological conditions [7, 8]. Therefore, antiresorptive drugs, such as bisphosphonates, receptor activator of nuclear factor-κB (RANK) ligand (RANKL) inhibitor, estrogen, or selective estrogen receptor modulators, are prevalent therapeutics that target osteolysis and rescue bone loss [11–13].

Recently, with the in-depth study in bone physiology, OCs, the giant (but not a fool), are manifesting more complex identities beyond their resorptive function. In particular, the reciprocal interactions between bone cells are attracting much attentions [14–16], because of the advanced understanding of the bone coupling between osteoclastic bone resorption and osteoblastic bone formation [3, 17, 18]. Through cell-cell contact, cell-bone matrix interaction, and paracrine factors, OCs have crosstalk with other bone cells, stem cells, and immune cells in the bone microenvironment, which affects recruitment, differentiation, and function of not only themselves but also the other cells [19–21]. This effect of OCs on other cells is more apparent during skeletal aging due to deteriorations on mesenchymal stem cell/mesenchymal stromal cell- (MSC-) derived osteogenesis and chondrogenesis, while HSC-derived osteoclastogenesis advances with increasing age, thereby gaining the initiative in the bone remodeling process and functioning predominantly over other factors [22–25]. It should be noted that OC-derived activities have both positive and negative effects, and those “pure” antiresorptive drugs (bisphosphonates or denosumab) for age-related bone disorder usually inhibit bone resorption with a concomitant reduction in bone formation owing to bone coupling, indicating the importance of OCs in maintaining normal bone remodeling after adulthood [11, 26, 27].

This review aimed to determine the essential roles of OC not just as a bone eater during bone remodeling but also as a positive contributor to the bone microenvironment and skeletal health. Specifically, we discuss how OCs contribute to the recruitment and differentiation of MSCs, as well as the following bone formation during remodeling. We hope this review can provide a different perspective on recognizing OCs when strategies are created to develop ideal therapeutic agents that target bone remodeling disorders characterized by excessive OC activity.

## 2. Osteoclasts and Bone Remodeling

Unlike bone modeling, which does not require coupled activities of OCs and OBs during skeletal growth and development, bone remodeling demands anatomically or spatially coupled activities of OCs and OBs to replace the old and damaged bone and to maintain calcium homeostasis in the body throughout one's life [28]. Each year, approximately 3 to 4 million basic multicellular units (BMUs) responsible for bone remodeling are initiated, and about 1 million of them are highly active as a standby for participating in bone turnover in the adult skeleton [28–30]. The remodeling process inside the BMUs does not occur randomly along the bone surface, but rather at specific sites, and it follows a well-orchestrated sequence of events that are typically divided into five stages: the activation of OC recruitment, initiation of osteoclastic bone resorption, transition from catabolism to anabolism due to OC apoptosis and OB recruitment, formation of the new organic matrix by OBs, and subsequent mineralization over time [28, 31]. In healthy adults, under physiological conditions, bone mass can be stable for one or two decades after reaching the peak volume due to a balance of the bone resorption and bone formation, that is, until age-related imbalance starts (heightened OC activity and reduced OB performance) [6, 22, 28].

OCs, the unique bone-resorbing cells, arise from HSCs and belong to the monocytic family [21, 32]. In the activation phase of bone remodeling, mononuclear OC precursors in the bone marrow or from blood circulation are attracted to prospective resorption sites, where they attach to the matrix surface and further differentiate into mature OCs (giant multinucleated cells) via cell fusion, termed as “multinucleation” [5, 32–34]. Mature OCs start to generate sealing zones on the targeted matrix surface during the resorption phase via the rearrangement of the cytoskeleton and the formation of a dense belt-like structure called the “actin ring” [35, 36]. The actin ring encloses the plasma membrane and makes it into a highly convoluted ruffled border which then serves as an exit site for protons and lysosomal proteases, such as cathepsin K (CTSK) to be secreted into the resorption lacunae, facilitating hydrolyzation and solubilization of the inorganic and organic components of bone [5, 20, 37]. By sensing the concentration of extracellular calcium [Ca^2+^]_o_ around the cell and responding to the change of intracellular Ca^2+^ concentration [Ca^2+^]_i_, OCs switch between the resorbing state featured by possessing actin rings and the nonresorbing/migrating state featured by scattered podosomes [38–40]. The resorbing activity of OCs gradually declines when basal [Ca^2+^]_i_ increases, whereas lower [Ca^2+^]_i_ reduces cell motility but enhances the anchoring capacity of the cell onto the bone matrix surface [38, 41]. Once resorption at one site is completed, OCs can move and start a new resorption cycle somewhere else or undergo apoptosis based on their lifespan [32, 36]. Among key molecules and signaling pathways involved in the process of osteoclastogenesis and resorption activity, RANK signaling is dominant through the entire life cycle of OCs and can be further amplified by costimulatory signals from immunoreceptor tyrosine-based activation motif- (ITAM-) associated immunoglobulin-like receptor (IgLR) signaling [38, 42–44]. Details of the RANK signaling network, along with other critical pathways that cooperate with it, such as calcium signaling pathway (Ca^2+^/calmodulin/calcineurin/NFATc1) and oxidative stress response pathway (ROS/Nrf2/Keap1), have been well summarized in several excellent papers and will not be discussed further in this review [32, 38, 45–47].

**Figure 1:**
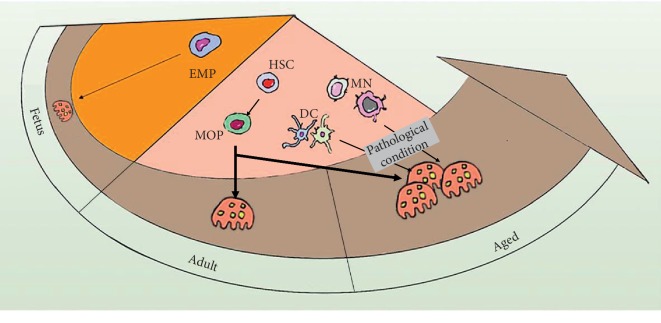
Origins of osteoclasts in an age-dependent manner [21]. Osteoclasts (OCs) differentiate from the embryonic erythro-myeloid progenitor (EMP) lineage during the embryonic and postnatal period. In adulthood, bone marrow myeloid/monocyte/macrophages (BMMs) derived from hematopoietic stem cells (HSCs) are the main origin of osteoclasts. Moreover, monocytes (MNs) and dendritic cells (DCs) are also important origins of osteoclasts in aged or pathological conditions. MOP: macrophage/osteoclast progenitor.

Recent advances widely explored the origins of OCs and associated them with aging and other pathological scenarios. It was not until the last decade that researchers started to decipher how aging affects the skeletal system tremendously. While osteogenic and chondrogenic differentiation from MSCs deteriorates, aging upgrades OC progenitors in both quality and quantity, including increased intrinsic expression of c-Fms and RANK, and enlarged OC progenitor pool [22–25]. As the origin of the OC progenitor, HSCs contribute to the reinforcement of the progenitors' pool by giving a bias toward myeloid development over lymphoid differentiation with increasing age [23, 48]. Madel et al. recently summarized different origins of OCs in an age-dependent manner (Figure 1): the embryonic erythro-myeloid progenitor (EMP) lineage during the embryonic and postnatal period, bone marrow myeloid/monocyte/macrophage (BMMs) lineage during adulthood, and conventional/mature monocytes (MNs), as well as dendritic cells (DCs) under inflammatory conditions which are usually seen in old age [21, 49]. In addition to the promotion of OC progenitors during aging, OC supporting cells, such as OBs, B cells, and T cells, also contribute to osteoclastogenesis by increasing RANKL expression and reducing osteoprotegerin (OPG) level in the bone microenvironment, although the population of these cells decreases with increasing age [24, 50–52]. Therefore, OC is vulnerable to be treated as a “foe” of skeletal health because of hyperactivity, especially in aged individuals. However, it does not negate its substantial role as a “friend” in removing the old and damaged bone, as well as a positive contributor during bone formation after adulthood, which has become more understandable in the last few years.

Several *in vitro* studies indicated that OC-derived factors directly affect MSC recruitment and OB differentiation [53–56]. Karsdal et al. reported that conditioned media (CM) from human OCs increased bone nodule formation in a dose-dependent manner, which was further confirmed by Kreja et al. [53, 54, 56]. Interestingly, they also found that the effect of OCs on MSC migration and OB differentiation can be independent of their resorption activity. Likewise, Henriksen's study indicated that mature OCs were sources of anabolic stimuli for OBs, and their interaction with the matrix can strongly affect the anabolic signals from OCs to OBs [55]. Conversely, a reduced number of OBs and bone formation were found in OC-poor osteopetrosis, indicating a critical role of OCs in regulating bone anabolic function [57]. All these findings suggest complex identities of the giant beyond the resorption function.

## 3. Osteoclasts and the Initiation of Bone Remodeling

The initiation phase of bone remodeling includes the recruitment of OC precursors, differentiation and functioning of OCs, and maintenance of bone resorption [28, 31]. The initiation of osteoclastogenesis largely depends on the crosstalk between OC precursors and the OB lineage cells. Emerging data supports the central regulatory role of osteocytes in the initial stage of bone remodeling [58–62]. As the most abundant cells in bone that are derived from OBs and embedded in the bone matrix, osteocytes play a role in determining which bone surface OCs are about to resorb [58, 59]. Through a network of osteocyte canaliculi, osteocytes can detect microfractures and microcracks in bone and contact other cells, such as OBs, on the bone surface. Bone fatigue induces apoptosis of osteocytes, which are localized to regions that contain microcracks, and this apoptosis was observed to precede OC invasion in the damaged area, which triggers subsequent bone remodeling in the targeted region [63].

Osteocytes have also crosstalk with OCs via secreted proteins. Osteocytes can control OC function by secreting RANKL and transforming growth factor beta (TGF-*β*) [64, 65]. RANKL, one of the essential osteoclastogenic factors, is mainly secreted by osteocytes [65–67]. Nakashima et al. [65] demonstrated that osteocytes express a much higher amount of RANKL and have a better capacity to support osteoclastogenesis than OBs and bone marrow stromal cells, which is a strong evidence for the crosstalk between osteocytes and OCs in bone remodeling. The MLO-Y4 osteocyte-like cell line represents a good model for studying the soluble interactions between osteocytes and OCs [64]. When mechanical scratching was applied to MLO-Y4 cells, enhanced secretion of osteoclastogenic factors, RANKL, and the monocyte colony-stimulating factor (M-CSF) was observed. The mechanical scratching of osteocytes induced the formation of tartrate-resistant acid phosphatase- (TRACP-) positive cells on top of the gel along the damaged region. No TRACP-positive cells were formed in the peripheral regions [59]. These findings indicate that soluble factors secreted from damaged osteocytes could locally induce and activate the initial phase of OCs formation.

The initiation of bone remodeling at the targeted bone site is essential for the renewal of an old or damaged bone matrix to prevent the skeleton from aging. Failure to trigger bone remodeling can result in accumulated microdamage and hypermineralization, which leads to reduced bone quality and increased fracture risk. Thus, retaining the crosstalk between OCs and osteocytes is beneficial for skeletal health when managing high turnover bone disorders, such as osteoporosis.

## 4. Effect of Osteoclasts on Mesenchymal Stem Cell Recruitment and Osteoblast Differentiation

After the old or damaged bone is resorbed by OCs, bone remodeling enters the second phase: the transition of OC to OB activity. In this reversal phase of bone remodeling, the microenvironment created by OC activity provides signals that aid in the cessation of bone resorption and the initiation of bone formation via the recruitment and differentiation of MSCs [17, 68]. The bone resorptive microenvironment is built by multiple factors that are released from the bone matrix during bone resorption or directly secreted by OCs locally, which also contribute to the establishment of the osteogenic microenvironment that promotes the recruitment of MSCs [4, 69–71]. MSCs are multipotent stem cells that are capable of differentiating into various cell types, such as OBs, adipocytes, and chondroblasts [72, 73]. In the bone marrow, MSCs are located around sinusoids and the perivascular network in the stroma [74, 75]. During bone remodeling and fractured-bone regeneration, MSCs migrate to the bone surface or fracture site and then differentiate into OBs to reconstruct the bone [76], subsequent to the osteoclastic resorptive phase. It has been well demonstrated that local growth factors and signals play important roles in the recruitment and commitment of MSCs [77], such as the bone morphogenetic protein (BMP) family [78], insulin-like growth factor (IGF) [79, 80], TGF-*β* [68, 81], fibroblast growth factor 2 (FGF-2) [82], vascular endothelial growth factors (VEGF) [78], and platelet-derived growth factors (PDGFs) [83, 84]. Moreover, emerging evidence showed that many of these local factors are associated with the viability and activity of OCs [17, 20, 54].

### 4.1. Osteoclastic Resorption Releases Bone Matrix Embedded Factors and Recruits Mesenchymal Stem Cells

Factors released from the bone matrix during bone resorption may be the first signal from OCs that has been found to affect MSCs. The bone matrix contains many latent growth factors that are deposited by OBs during matrix construction and then released by osteoclastic resorption on the bone surface [85, 86]. Howard et al. [87] firstly proposed that the release of coupling factors embedded in the bone matrix may positively affect MSC-derived osteogenesis. To date, several matrix-derived factors have been identified as potential factors involved in bone remodeling, such as TGF-*β* [85, 88], IGF-1 [69], bone morphogenetic protein (BMP)-2 [89, 90], and vascular endothelial growth factor (VEGF) [91]. In particular, matrix-derived TGF-*β*1 and IGF-1 have shown definite effects linking bone resorption to MSC recruitment and differentiation based on genetically manipulated mice data. Tang et al. [88] demonstrated that TGF-*β*1 released during OCs culture on bone slices *in vitro* induces the migration of MSCs. They also found high levels of active TGF-*β*1 in the bone resorption-conditioned media (BRCM) when functional OCs were cultured with bone slices *in vitro*, whereas active TGF-*β*1 was barely detectable in the conditioned media prepared without bone slices. Moreover, BRCM prepared using OCs generated from normal mice and bone slices prepared from TGF-*β*1 1 knockout (TGF-*β*1−/−) mice was significantly less effective in promoting the migration of BMSCs [88], demonstrating that matrix-derived TGF-*β*1 plays a key role in recruiting MSCs. Similarly, it has also been well demonstrated that IGF-1 released from the bone matrix by functioning OCs stimulated OB differentiation of MSCs by activating the mammalian target of rapamycin (mTOR) through the PI3K-Akt pathway [69].

### 4.2. Osteoclast-Secreted Factors Recruit Mesenchymal Stem Cells and Promote Osteoblast Differentiation

Besides the matrix-derived factors, increasing data also suggest that factors directly secreted by OC lineage cells play a crucial role in coupling osteoclastic bone resorption with osteoblastic bone formation. Henriksen et al. [55] performed a research to address the anabolic effect of OC linage cells in different stages. They collected the conditioned medium (CM) from macrophages, pre-OCs, and mature functional or nonresorbing OCs and tested their effects on osteogenesis *in vitro*. Their results suggested that CM from macrophages did not induce bone formation, while CM from mature OCs promoted osteogenesis, both dependent on and independent of their resorptive activity. Kim et al. [56] also conducted a research to explore when the coupling factors are taking effect during osteoclastogenesis. They found that CM from OCs in the early stage of differentiation predominantly enhanced the migration of osteoblastic lineage cells, confirming that OCs play an important role in the coupling by stimulating pre-OBs migration.

To date, increasing studies have identified numerous secreted molecules from OCs and explored their potential roles in bone remodeling. In Table 1, we have summarized the OC-secreted factors and their effects on MSC migration, OB differentiation *in vitro*, or bone formation *in vivo*. Among them, factors including Afamin [56], CXCL16 [98], PDGF-BB [101, 102], and S1P [104, 105] secreted by OCs can promote the migration of MSC or OB progenitors, and factors such as BMP6 [98], C3a [95], CT-1 [96], CTHCR1 [97], HGF [99, 100], SLIT3 [107], Trap [108, 109], and vesicular RANK [110] exhibit enhancing effects on OB differentiation *in vitro* or bone formation *in vivo*. However, some other factors such as Sema4D [111], sclerostin [112], and exosomal miR-214-3p [113] show an inhibiting effect on bone formation. These factors may act as a “fine-tuning” mediator of the bone remodeling process in the BMUs, by inhibiting the remodeling process under some special conditions. Besides, these factors are often highly expressed in OCs from aged or ovariectomized mice, suggesting that they may play a role in bone remodeling disorders during aging. Overall, on the basis of the current findings, most OC-secreted factors show enhancing effects on MSC recruitment or OB differentiation, indicating an essential role of OCs in maintaining normal bone formation during the remodeling process.

### 4.3. Osteoclast and Osteoblast Cell-Cell Contact: A Potential Mechanism of Transition in Bone Remodeling

OCs and OB lineage cells can also communicate through cell-cell contact to achieve the coupling of bone resorption and formation. Traditionally, it has been thought that OCs and OBs do not occur simultaneously at the same BMUs, and direct contact between mature OBs and functioning OCs is relatively rare [115]. In recent years, direct OC–OB contact *in vivo* has been detected using transmission electron microscopy [31] and intravital two-photon imaging [116]. Furuya et al. demonstrated that mature OCs became nonresorptive when they made contact with mature OBs, and intermittent administration of the parathyroid hormone (PTH) led to an increase in cell-cell contact between OCs and OBs, which causes bone anabolic effects [116].

**Figure 2:**
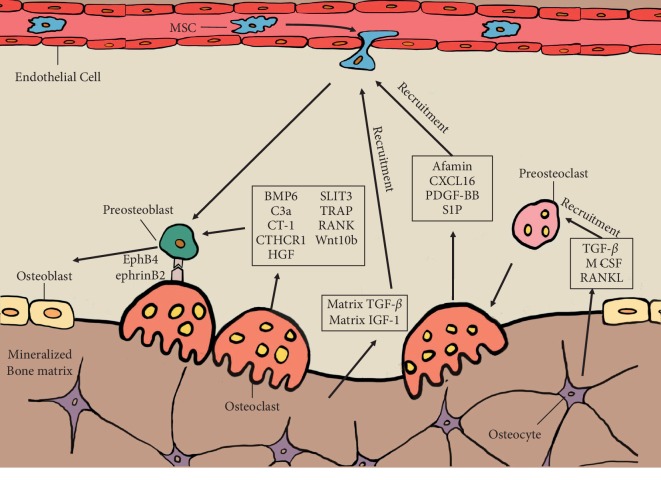
Schematic illustration of the interaction between osteoclast (OC) and osteoblast (OB) lineage cells in bone remodeling. OC precursors are activated by TGF-*β*, M-CSF, and RANKL secreted by osteocytes and attracted to prospective resorption sites. Once attached to the bone matrix, OC precursors can differentiate into mature OCs. Mature OCs will further acidify and resorb the mineralized bone matrix by pumping hydrogen ions into resorptive captivity through their ruffled border structure. During bone resorption, OC can release several coupling factors, such as matrix-derived TGF-*β*, matrix-derived IGF-1, Afamin, CXCL16, PDGF-BB, and S1P et al., which recruit circulated mesenchymal stem cells (MSCs) to the resorption area. Besides, OC also secretes some other coupling factors, such as BMP6, SLIT3, C3a, TRAP, CT-1, and RANK et al., which further promote the differentiation from MSCs towards OBs. Additionally, the ephrinB2/ephB4 interaction between OC and OB precursors suppresses the bone resorption activity of OCs, whereas such interaction could trigger OB differentiation of OB precursors and enhance bone formation.

How does the cell-cell contact cause bidirectional effects between OCs and OBs? EphrinB2/EphB4 interaction between OCs and OBs plays a role in the transition from bone resorption to the formation. Ephrin/Eph family members are local mediators of cell function through contact-dependent manner during various developmental processes [117, 118]. Interaction between ephrin-expressing and Eph-expressing cells leads to bidirectional signal transduction. Mature OCs express ephrinB2, whereas OB precursors express EphB4 (Figure 2). Forward signaling through the EphB4 receptor into OB precursors enhances osteogenic differentiation by reducing RhoA activity, while reverse signaling through ephrinB2 ligand into OCs suppresses OC function by inhibiting the osteoclastogenic c-Fos-NFATc1 cascade [119]. However, it has also been suggested that mice lacking ephrinB2 showed no skeletal abnormalities [119]. Thus, the role of ephrinB2/EphB4 interaction between OCs and OBs in the transition from bone resorption to formation needs further confirmation.

## 5. Summary and Perspectives

The skeletal system provides mechanical support, protects vital organs, and controls mineral homeostasis in the human body. It is the constant bone remodeling throughout one's life that removes the old and damaged bone, keeping the skeletal system healthy. During the recent decade, many studies have demonstrated mechanisms for how osteoclastic bone resorption contributes to the subsequent bone formation in bone remodeling (Figure 2) and provided a well-rounded understanding of the roles of OCs in maintaining proper bone remodeling.

Osteoporosis, the most prevalent disorder of bone remodeling by far, is characterized by the heightened activity of OCs [6, 7]. Currently, the available treatments of osteoporosis comprise antiresorptive agents, such as bisphosphonate and denosumab, and anabolic treatments such as PTH [6, 13]. However, most antiresorptive agents that suppress OC differentiation will concomitantly impair bone formation because of the coupling effect, leading to an unsatisfactory long-term effect and potentially increasing the likelihood of long-term adverse events, such as osteonecrosis of the jaw [120]. Thus, new agents under development for osteoporosis may try to retain the OC coupling factors while inhibiting OC functions. Odanacatib, a small-molecule inhibitor of CTSK, can decrease bone resorption without affecting OBs and appears to promote bone formation [106, 121, 122], probably because of the suppression on OC activity rather than the inhibition on OC viability, thus allowing continuous crosstalk between OCs and OBs. Unfortunately, because of the unforeseen cerebrovascular events, the clinical development of odanacatib was terminated. The side effects may result from the off-target effects of CTSK inhibitors on other members of the cathepsin family, such as cathepsins B, L, and S. Nonetheless, the experience learned from the underlying biology of CTSK inhibitors could guide future therapeutic approaches for osteoporosis: dissociating the inhibition of bone resorption from the coupled reduction in bone formation. This may be a promising strategy in the development of a new drug and we believe that a better outcome will be achieved when proper strategies are made to coordinate OCs and OBs in managing bone remodeling disorders.

## Figures and Tables

**Table 1:** Summary of osteoclast-secreted factors on bone remodeling.

Factor secreted by osteoclasts		Effect on bone remodeling	Reference
*Osteoclast-derived enhancing factors of bone formation*			
Afamin	Afamin	Afamin secreted by osteoclasts in the early stage of differentiation stimulates preosteoblasts migration *in vitro* via the Akt-signaling pathway Afamin can prevent Wnt proteins from aggregating and deliver Wnt ligands to its receptors on the cell surface, which plays an important role in osteogenesis	[56, 92]
BMP6	Bone morphogenic protein 6	Synthesis of BMPs has been confirmed in osteoclasts using immunocytochemistry and in situ hybridization BMP6 promotes osteoblast differentiation	[93, 94]
C3a	Complement component 3a	C3 gene expression increases during osteoclastogenesis, and the cleavage product C3a is detected in the conditioned medium of osteoclasts C3a promotes osteoblast differentiation	[95]
CT-1	Cardiotrophin-1	CT-1 promotes osteoblast differentiation Neonatal Ct-1−/− mice have decreased osteoblast numbers and BV/TV	[96]
CTHCR1	Collagen triple repeat containing1	CTHCR1 is secreted by mature bone-resorbing osteoclasts CTHCR1 stimulates osteoblast differentiation Osteoclast-specific deletion of CTHCR1 in mice resulted in osteopenia due to reduced bone formation	[97]
CXCL16	Chemokine (C-X-C motif) ligand 16	TGF-*β*1 released from the bone matrix during bone resorption induces CXCL16 production in osteoclasts, which promotes migration of osteoblast progenitors in bone remodeling	[98]
HGF	Hepatocyte growth factor	Osteoclasts can synthesize and secrete biologically active HGF, which promotes osteoblast proliferation and increases osteopontin expression in osteoblasts	[99, 100]
PDGF-BB	Platelet-derived growth factor BB	PDGF-BB induces MSC migration, but it inhibits osteoblast differentiation	[53, 101–103]
S1P	Sphingosine-1-phosphate	S1P stimulates MSC migration and promotes osteoblast differentiation Raising S1P levels in adult mice markedly increased bone formation	[104–106]
SLIT3	slit guidance ligand 3	Osteoclast-secreted SLIT3 synchronously inhibits bone resorption and stimulates bone formation SLIT3 injection in mice markedly rescued bone loss after ovariectomy surgery	[107]
TRAP	Tartrate-resistant acid phosphatase	TRAP promotes osteoblast differentiation TRAP overexpressing transgenic mice have an increased rate of bone turnover	[108, 109]
Vesicular RANK	Vesicular TNF receptor superfamily member 11A	Mature OCs secrete vesicular RANK, which binds osteoblastic RANKL and promotes bone formation via triggering RANKL reverse signaling	[110]
Wnt10b	Wnt family member 10b	Wnt10b expression increases during osteoclastogenesis Wnt10b promotes mineralization	[104]

*Osteoclast-derived inhibiting factors of bone formation*			
LIF	Leukemia inhibitor factor	LIF inhibits TGFb1-induced osteoblast migration	[98]
Sema4D	Semaphorin 4D	Sema4D suppresses bone formation by inhibiting IGF-1 signaling Sema4d-/- mice show an osteosclerotic phenotype due to augmented bone formation	[111]
SOST	Sclerostin	SOST is expressed in osteoclasts from aged mice and inhibits osteoclast-mediated stimulation of mineralization	[112]
Exosomal miR-214-3p	Exosomal miR-214-3p	miR-214-3p reduces bone formation in elderly women with fractures and in ovariectomized mice	[113, 114]
